# Direct visualization of interfacial defect effects on polarization switching in BaTiO_3_ tunnel junctions

**DOI:** 10.1126/sciadv.aeh5401

**Published:** 2026-09-16

**Authors:** Jeehun Jeong, Jinho Byun, Xiaoshan Xu, Alexei Gruverman, Jaekwang Lee, Sang Ho Oh

**Affiliations:** ^1^ Department of Energy Engineering, KENTECH Institute for Energy Materials and Devices, Korea Institute of Energy Technology (KENTECH), Naju 58330, Republic of Korea.; ^2^ Department of Physics and Astronomy, University of Nebraska, Lincoln, NE 68588, USA.; ^3^ Department of Physics, Pusan National University, Busan 46241, Republic of Korea.

## Abstract

Deterministic control of polarization switching at complex oxide interfaces is essential for high-performance ferroelectric devices, yet the microscopic competition between external fields and polarization response remains difficult to probe directly. Combining atomic-scale scanning transmission electron microscopy and electron energy loss spectroscopy with in-situ biasing, we establish an asymmetric interfacial pinning mechanism in epitaxial Pt/BaTiO_3_/La_2/3_Sr_1/3_MnO_3_ ferroelectric tunnel junctions. At the Pt/BaTiO_3_ interface, an oxygen vacancy–rich pinning layer induces Ti reduction and a strong, uniform downward electric field. In contrast, the BaTiO_3_/La_2/3_Sr_1/3_MnO_3_ boundary is characterized by localized La_Mn_ antisite defects that generate internal fields through localized tensile strain. Under an upward external field, this competitive landscape forces the formation of a stable, head-to-head domain wall within the 3-nanometer-thick BaTiO_3_ barrier, preventing the system from reaching a homogeneous polarization state. Our findings demonstrate that ferroelectric reversibility is fundamentally constrained by a mutual stabilization of cation and anion defects, providing a framework for engineering electrode interfaces at the limit of unit-cell thickness.

## INTRODUCTION

The ability to manipulate ferroelectric polarization at the unit-cell level is a cornerstone of modern condensed matter research (*1*–*3*) and the primary driver for next-generation nonvolatile memory (*4*, *5*). Ferroelectric tunnel junctions (FTJs) capitalize on this by using an ultrathin ferroelectric barrier to modulate electron tunneling, creating distinct resistive states for logic-in-memory (*1*, *5*, *6*) and neuromorphic applications (*7*, *8*). In a typical FTJ, the tunneling electroresistance (TER) effect is governed by how polarization reversal reshapes the potential landscape (*9*). However, as the ferroelectric barriers are scaled down to the ultrathin limit, their functional properties become entirely dictated by the complex energy landscape of the electrode interfaces (*10*, *11*).

At the ultrathin limit of ferroelectrics, the electrode/ferroelectric interfaces are the governing elements that either amplify or suppress device performance. Factors such as finite metal screening (*1*, *12*, *13*), interface dipoles (*14*–*16*), and local stoichiometry (*17*–*19*) establish boundary conditions that affect the initial polarization and the subsequent switching pathways. In ultrathin ferroelectric heterostructures, interface-driven boundary conditions can generate preferential polarization states and asymmetric switching characteristics. In BaTiO_3_-based heterostructures, initial downward-preferred polarization states and asymmetric switching behaviors have been observed through electrical characterization (*20*–*28*) and have been attributed to oxygen vacancy–related interfacial pinning (*20*–*23*), work-function asymmetry between dissimilar electrodes (*24*–*26*), and strain gradients (*27*, *28*). Recent studies have further highlighted the important role of bottom-interface chemistry and electrostatics in stabilizing preferential polarization states (*29*, *30*). In addition, substrate-mediated strain can strongly influence the energetic stability of polarization configurations in ultrathin ferroelectric heterostructures (*31*). However, despite extensive electrical characterization and theoretical discussions, direct evidence identifying how interfacial defect structures stabilize preferential polarization states has remained elusive.

At the atomic scale, the impact of atomic-scale imperfections is amplified; specifically, point defects—such as oxygen vacancies (V_O_s) (*19*, *32*–*36*) and cation antisites (A-on-B or B-on-A substitutions) (*37*–*40*)—emerge as determinants of both transport and polarization dynamics. The V_O_s introduce localized gap states that modulate the effective barrier height (*41*, *42*) and alter space-charge profiles (*32*, *43*, *44*). In parallel, cation antisites can distort the BO_6_ octahedra, inducing large local strains that couple to the ferroelectric order parameter (*37*–*40*). Despite their importance, directly visualizing how defect-mediated local fields interact with externally applied electric fields at the atomic scale remains challenging. This knowledge gap is essential to bridge to overcome incomplete polarization reversal, where a system remains trapped in a nonhomogeneous state despite a strong external driving force.

Here, we investigate these defect-interface couplings to directly visualize this competition in a Pt/BaTiO_3_ (BTO)/La_2/3_Sr_1/3_MnO_3_ (LSMO) metal-ferroelectric-metal (MFM) FTJ system. By combining unit-cell–resolved scanning transmission electron microscopy (STEM) and electron energy-loss spectroscopy (EELS) with in-situ biasing, we reveal a dual-interface pinning landscape. We show that noble-metal/oxide redox at the Pt/BTO interface creates a V_O_-enriched pinning layer, while La_Mn_ antisite formation at the BTO/LSMO interface generates localized internal fields. This interfacial defect environment forces the formation of an overall head-to-head domain configuration within the 3-nm-thick BTO barrier, preventing a transition to a homogeneous state. Supported by density functional theory (DFT), our findings establish that the mutual stabilization of cation and anion defects sets a fundamental constraint on ferroelectric reversibility at the unit-cell limit.

## RESULTS

### Device structure and initial polarization

**Fig. 1.**
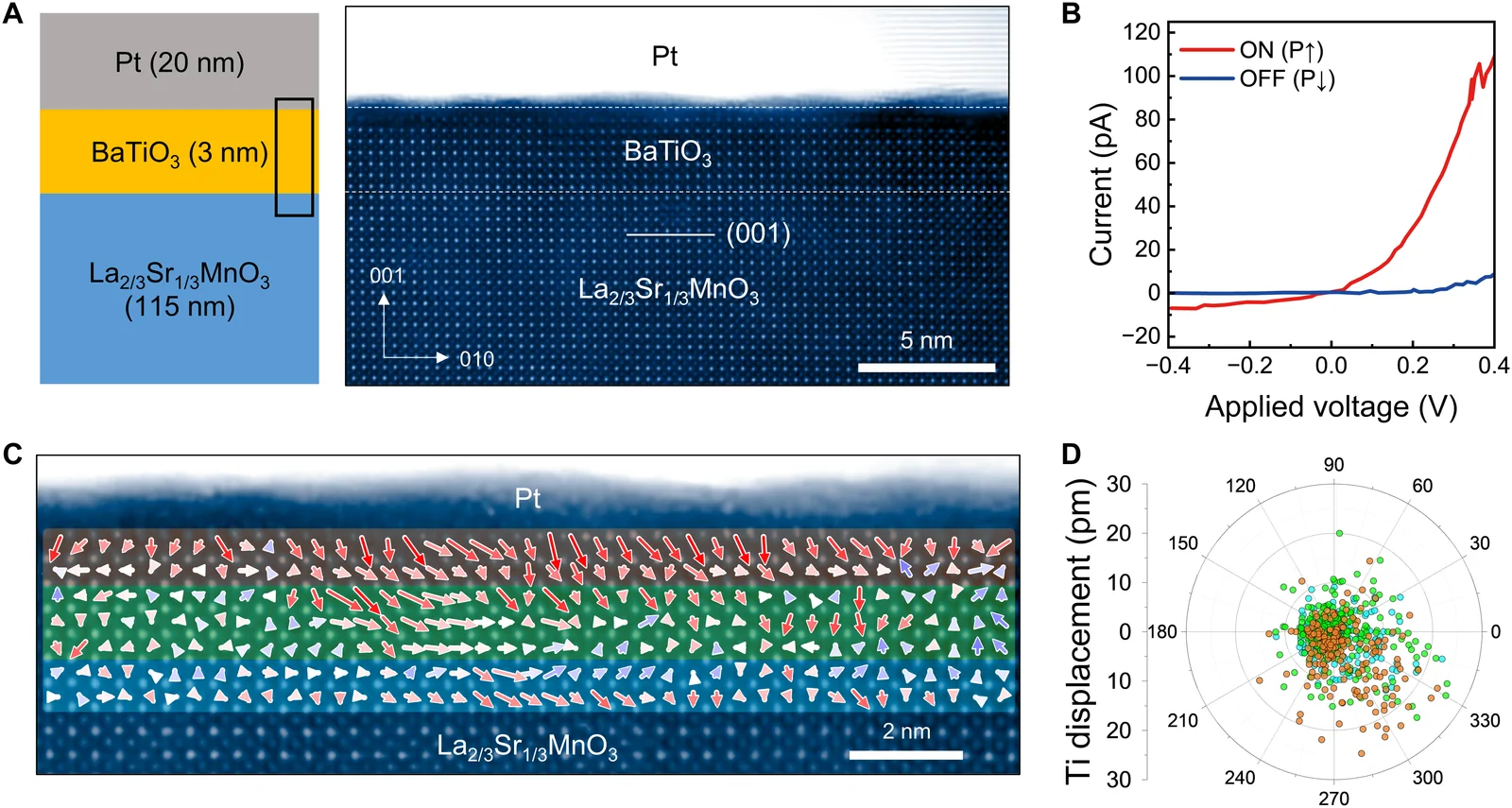
Structure and initial polarization state of the Pt/BTO/LSMO FTJ. (**A**) Cross-sectional schematic of the Pt/BTO (8 unit cells)/LSMO FTJ used for the in situ STEM biasing study, together with a representative atomic-resolution HAADF-STEM image of the device. (**B**) Measured current-voltage (*I*-*V*) characteristics of the FTJ in the ON and OFF resistance states, corresponding to opposite polarization directions. The applied write voltages were ±3 V. (**C**) Atomic-resolution HAADF-STEM image of the FTJ in the as-prepared state, with Ti displacement vectors overlaid to visualize the magnitude and direction of Ti off-centering within the BTO layer. (**D**) Polar plot of the Ti displacement vectors extracted from (C). The colors in the polar plot correspond to the spatial regions indicated by the same colors in the displacement map in (C). Despite the broad scatter, the Ti-displacement vectors preferentially populate the sector between the rightward in-plane direction and the downward direction, consistent with a polarization configuration that combines in-plane rotation with a net downward orientation.

Epitaxial Pt/BTO/LSMO heterostructures were fabricated on a (001) SrTiO_3_ (STO) substrate (Fig. 1A). The ferroelectric barrier is an 8–unit cell BTO layer deposited on a 115-nm-thick LSMO bottom electrode (BE), and a 100-nm-thick Pt top electrode (TE) completes the FTJ stack. Before TE deposition, piezoresponse force microscopy (PFM) on the as-grown BTO confirmed robust ferroelectricity. Local writing with ±3 V pulses, chosen such that |*V*_write_| exceeds the coercive voltage |*V*_c_| determined from the PFM hysteresis, produced stable up/down domains. After Pt deposition, low-bias *I*-*V* sweeps from −0.4 to +0.4 V recorded two distinct resistance states associated with opposite polarization orientations, yielding an ON/OFF ratio of 68 (Fig. 1B). The measured TER indicates that two resistance states associated with opposite polarization orientations are effectively switched or stabilized within the junction. However, because the TER extracted from low-bias *I*-*V* sweeps provides only a junction-averaged electrical signature, direct investigation of the local polarization response and the resulting internal domain configuration is necessary to reveal the microscopic (e.g., interfacial/defect-related) factors that limit further improvement.

To structurally register the BTO barrier and the interfaces with electrodes, cross-sectional lamellae were prepared by focused ion beam (FIB) milling and examined by STEM-EELS (fig. S1). Elemental mapping showed a TiO_2_ termination at the BTO/LSMO interface and a BaO termination at the BTO/Pt interface.

We quantified the local polarization by measuring Ti-site displacements from the centrosymmetric position of Ba sublattice as references in high-angle annular dark-field (HAADF) STEM images. We note that oxygen columns are not directly visible in HAADF-STEM images of these relatively thick FIB lamellae (∼60 to 80 nm) prepared for in situ biasing, so quantitative polarization values are inferred from the Ti displacements. The polarization vectors exhibit a pronounced asymmetry across the BTO barrier: near the Pt interface, polarizations are large and point downward; toward the interior, amplitudes decrease and vectors rotate partially in-plane; near the LSMO interface, the net polarization again points downward (Fig. 1C). This pattern implies a built-in asymmetry that favors the polarization-down state (P↓) even before biasing, consistent with a pinned layer at the Pt side and a weaker but nonnegligible electric field at the LSMO side.

### In situ switching: Asymmetric response and head-to-head domain formation

**Fig. 2.**
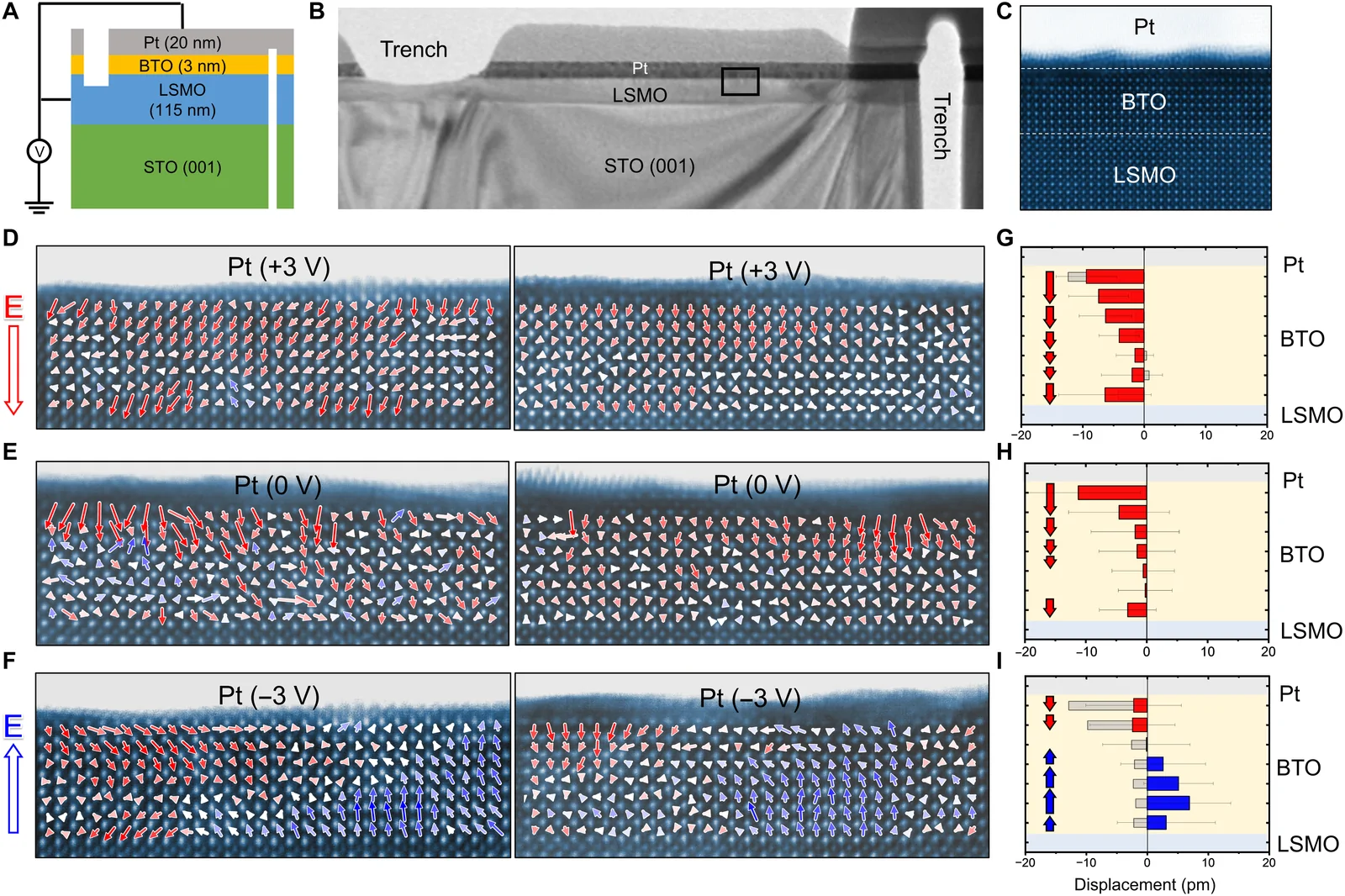
Bias-dependent polarization switching in the FTJ. (**A**) Schematic illustration of the TEM sample geometry for in situ biasing experiments. (**B**) Low-magnification TEM image of the FIB-prepared lamella used for in situ STEM measurements. (**C**) Atomic-resolution HAADF STEM image of the Pt/BTO/LSMO FTJ. (**D** to **F**) Atomic-resolution HAADF-STEM images of the BTO barrier acquired under +3 V (D), 0 V (E), and −3 V (F) bias, respectively. Ti displacement vectors are overlaid on the Ti-O atomic columns. For each applied bias, two sets of results obtained from different locations are shown. (**G** to **I**) In-plane averaged Ti displacement profiles corresponding to (D) to (F). Error bars represent the SD. The Ti displacement measured in the unbiased state (gray dataset) serves as a reference for those measured under applied biases. The colored arrows denote the magnitude and direction of polarization evolution under applied bias.

We then applied ±3 V to the Pt electrode in situ while grounding LSMO (Fig. 2, A to C). Under +3 V (field pointing downward), the preexisting P↓ configuration persists with little change near the Pt side, indicating that this interfacial region is already aligned with the field and strongly pinned (Fig. 2D), preserving the device’s high-resistance state. In contrast, under −3 V (field pointing upward), the LSMO-proximal layers partially reverse to P↑, but a several unit-cell-thick region adjacent to Pt resists reversal. This incomplete switching leaves a head-to-head wall configuration with a compensated central zone where polarization magnitudes diminish or rotate in-plane (Fig. 2F). Layer-averaged out-of-plane polarization profiles quantify this asymmetric switching behavior: near Pt, a residual downward component persists under −3 V, whereas the LSMO side more readily tracks the applied field (Fig. 2, G to I). This bias-dependent asymmetry, likely stemming from interfacial constraints during fabrication of the FTJ device, is therefore fundamental to understanding the memristive mechanism and dictates the attainable resistive contrast in these devices.

### Oxygen-vacancy accumulation and Ti reduction at the Pt/BTO interface

**Fig. 3.**
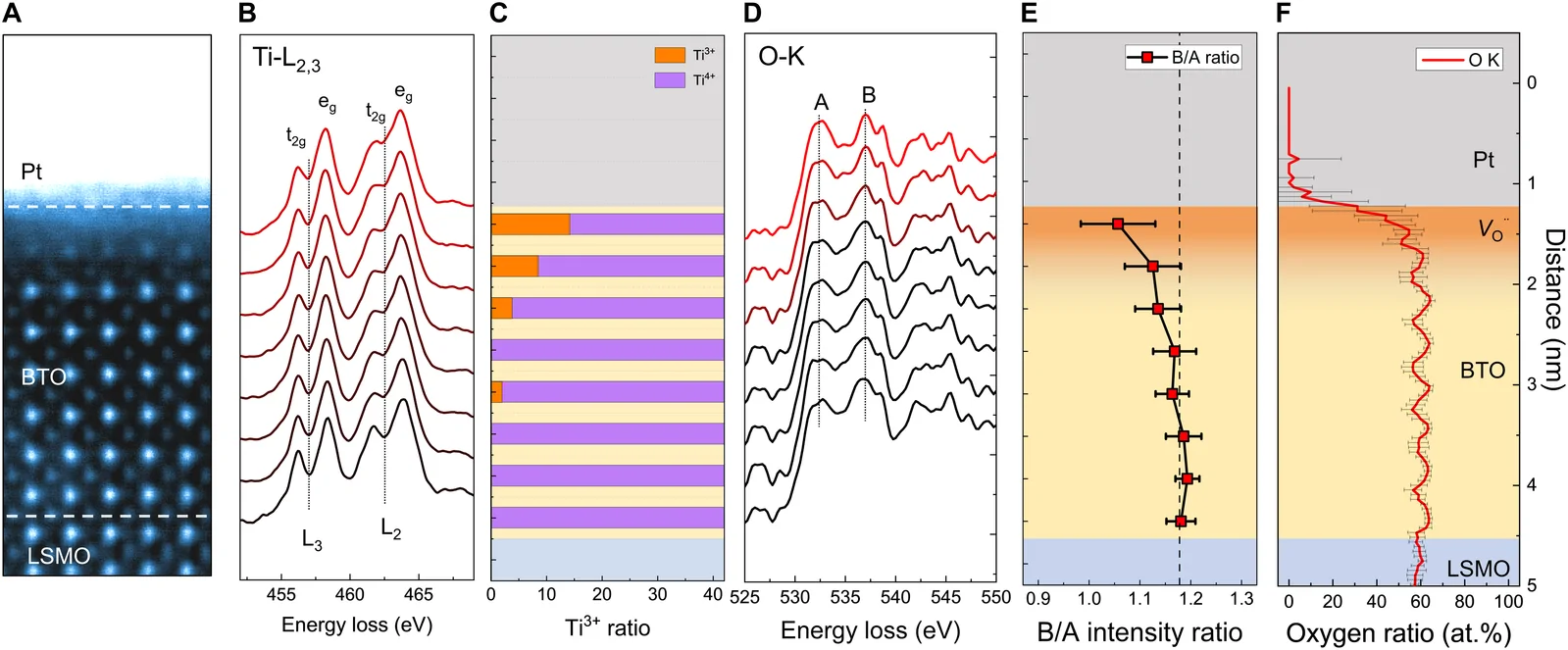
Oxygen vacancies detected by STEM-EELS near the Pt/BTO interface. (**A**) ADF-STEM survey image acquired for EELS measurement. Ti-L_2,3_ and O-K edge EEL spectra extracted from each TiO_2_ layer across the BTO barrier are displayed. (**B**) Ti-L_2,3_ edge EEL spectra. The interfacial spectra near Pt/BTO interface (red) exhibit modified fine structures associated with Ti^3+^ states. (**C**) Ti^3+^/Ti^4+^ ratio determined by MLLS fitting. (**D**) O-K edge EEL spectra. The first peak (labeled as A) corresponds to O 1*s* → O 2*p*–Ti 3*d* hybridized states, whereas the second peak (labeled as B) corresponds to O 1*s* → O 2*p*–Ba 5*d* hybridized states. The reduced intensity of peak B near Pt/BTO interface indicates oxygen deficiency. (**E**) Intensity ratio of peak B to A. The decreased B/A intensity ratios near the interface indicate the presence of V_O_. (**F**) Oxygen atomic concentration determined from the integrated O-K, Ti-L_2,3_, and Ba-M_4,5_ edges. Oxygen concentrations below stoichiometric value of 60% confirm interfacial oxygen deficiency.

To identify the chemical origin of the pinning layer, we performed spatially resolved EELS across the BTO (Fig. 3). The Ti-L_2,3_ edges exhibit clear t_2g_-e_g_ splitting in the BTO interior and near the LSMO interface; however, this splitting diminishes toward the Pt interface, consistent with partial Ti reduction (Fig. 3, A and B) (*45*). Multiple linear least squares (MLLS) decomposition reveals a notable increase in the Ti^3+^/Ti^4+^ fraction confined to the top 1 to 2 unit cells near the Pt electrode (Fig. 3C).

Concurrently, the O-K edge fine structure displays distinct modifications near the Pt interface (Fig. 3, D and E). The first peak (labeled as A), corresponding to the transition from O 1*s* to hybridized O 2*p*–Ti 3*d* orbitals, remains consistent, whereas the second peak (labeled as B), originating from the transition to hybridized O 2*p*–Ba 5*d* orbitals, exhibits a marked reduction in intensity (*45*, *46*). This selective attenuation of peak B serves as a well-established feature of V_O_ and associated local structural distortion at the interface. Furthermore, the atomic concentration of oxygen drops below the stoichiometric reference of 60% at the interfacial layer (Fig. 3F). These features collectively identify a continuous, V_O_-rich pinning layer that remains robust even under electric fields of ∼10^7^ V cm^−1^.

The accumulation of interfacial V_O_s is consistent with previous reports on metal/ferroelectric heterostructures (*44*, *47*–*49*), where V_O_ redistribution and interfacial redox reactions are frequently driven by electrochemical potentials at the metal/ferroelectric interface. Serving as a permanent pinning site, this V_O_-enriched layer anchors the polarization in a downward orientation (P↓) (*50*, *51*), effectively constraining the polarization state even under high external bias. The robust anchoring of polarization near the Pt/BTO interface thus prevents effective modulation of the tunneling barrier height, contributing to the suppressed TER ratio and the observed asymmetry in switching behavior.

**Fig. 4.**
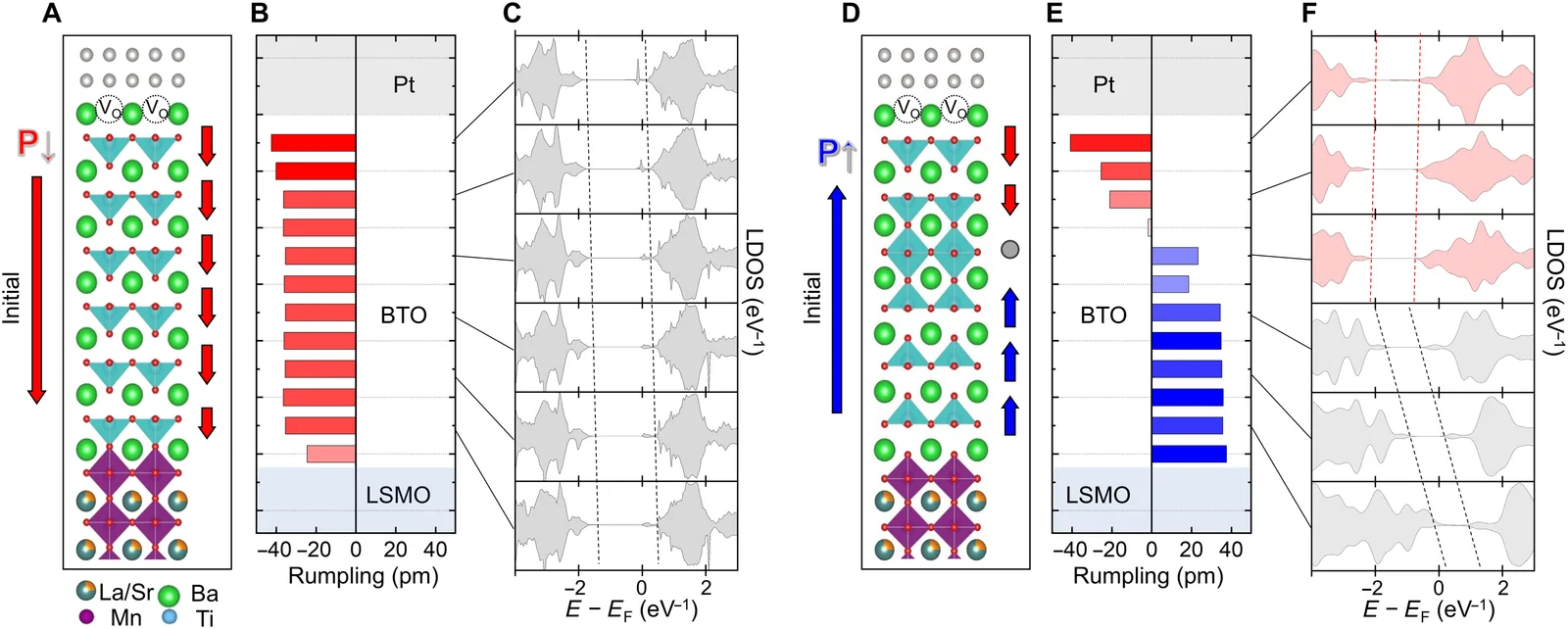
DFT calculations of polarization pinning by oxygen vacancies at the Pt/BTO interface. (**A** and **D**) Relaxed atomic structures initialized in the (A) polarization-down (P↓) and (D) polarization-up (P↑) states. The polarization of each layer is indicated by a colored arrow next to the layer, showing (A) a stable P↓ state where the positive space charge of the V_O_ layer is screened by the negative bound charge, and (D) the P↑ state exhibiting a head-to-head polarization configuration. (**B** and **E**) Calculated layer-by-layer polarization profiles assessed by measuring rumpling amplitude (relative displacement between cations and oxygen planes) for the (B) P↓ and (E) P↑ states. In (E), while the interior BTO layers switch to upward polarization (blue bars), the layers adjacent to the Pt interface remain pinned downward (red bars) due to the V_O_ defects, resulting in a head-to-head domain configuration. (**C** and **F**) Layer-resolved density of states (LDOS) of the Pt/BTO/LSMO heterostructure calculated for the (A) P↓ and (D) P↑ states. The dashed lines guide the conduction band minimum (CBM) and valence band maximum (VBM) profiles, indicating the direction of the internal electric field.

The influence of V_O_s present near the Pt TE on the switching behavior of polarization was investigated by carrying out DFT calculations. Electrostatically, donor-like V_O_s generate a positive space charge that is best screened by negative bound charge at the top interface, which is realized when the polarization points downward (Fig. 4, A to C). When an upward external field is applied, electrons from the V_O_ layer are trapped in shallow states at the center of the BTO film, altering the barrier profile to a head-to-head–like profile. Both effects cooperate to stabilize P↓ near Pt and to reduce the driving force for reversal when the external field is upward (Fig. 4, D to F), explaining the observed incomplete switching.

### Antisite defect-mediated polarization pinning at the BTO/LSMO interface

**Fig. 5.**
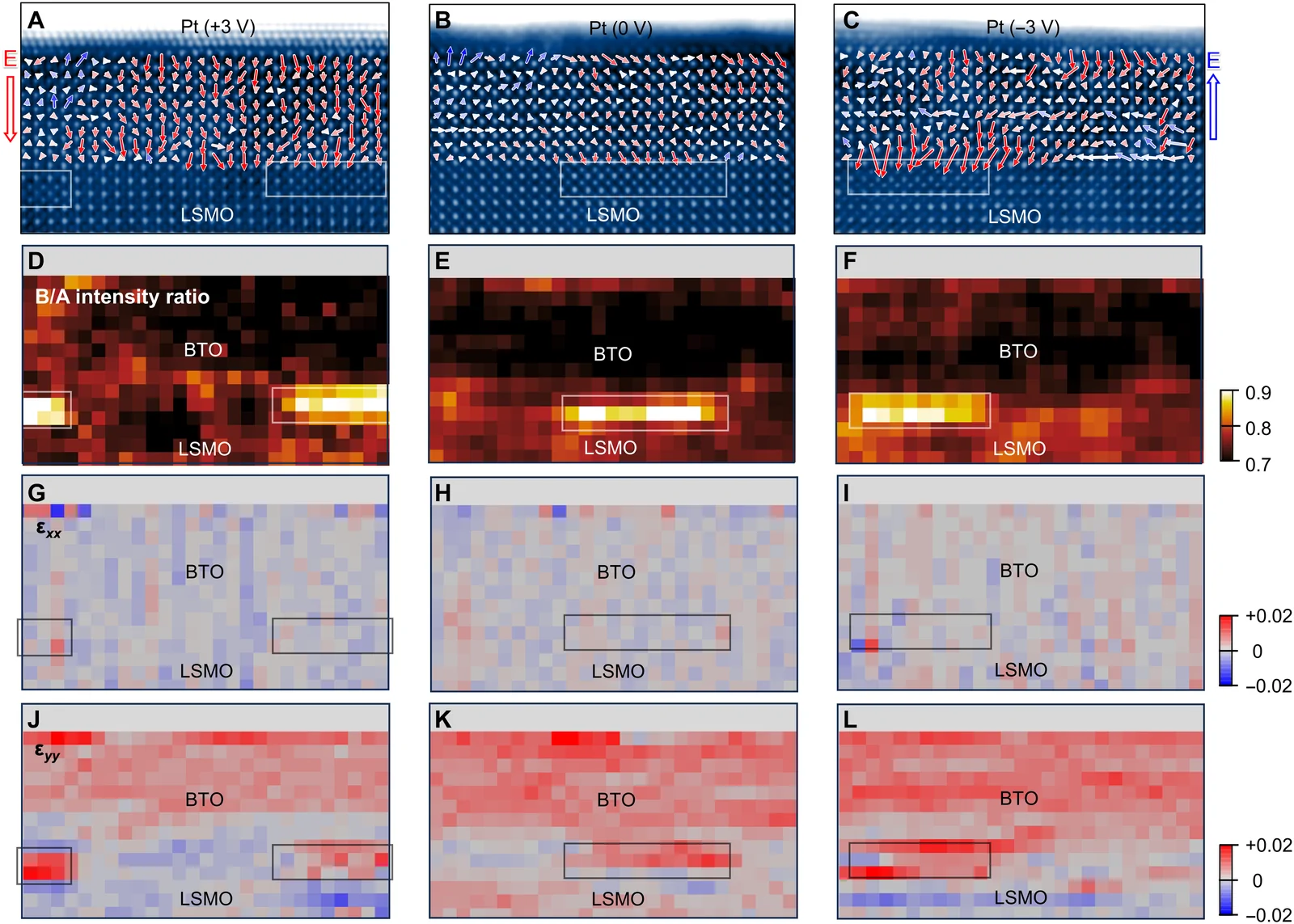
Polarization pinning and local strain associated with antisite defects near the BTO/LSMO interface. (**A** to **C**) Atomic-resolution HAADF-STEM images of the BTO film in the Pt/BTO/LSMO FTJ acquired under +3 V (A), 0 V (B), and −3 V (C) bias, showing defects within the LSMO electrode. Ti displacement vectors are overlaid on the Ti-O columns. (**D** to **F**) Corresponding B-site/A-site normalized HAADF intensity ratio maps obtained under +3 V (D), 0 V (E), and −3 V (F) bias. Elevated intensity ratios near the BTO/LSMO interface (white rectangles) indicate La_Mn_ antisite defects. These regions retain a downward Ti displacement under applied field, indicative of interfacial polarization pinning. (**G** to **I**) In-plane strain (ε*_xx_*) maps extracted from the same regions, showing relatively weak strain variation near La_Mn_ antisite-rich regions. (**J** to **L**) Out-of-plane strain (ε*_yy_*) maps extracted from the same regions, revealing localized tensile strain concentrated around the La_Mn_ antisite-rich interfacial areas.

In addition to the uniform pinning layer at the Pt/BTO interface, we identify localized regions adjacent to the LSMO electrode that exhibit a persistent downward polarization. These zones remain field-irresponsive regardless of the applied bias, suggesting the presence of discrete interfacial pinning centers (Fig. 5).

HAADF-STEM images reveal atomic-scale Z-contrast anomalies on selected B-site columns within LSMO (Fig. 5, A to C). Because Z-contrast HAADF intensity increases with the average atomic number of a column, these bright B-sites suggest cation species heavier than Mn occupying nominal B positions. To quantify this, we computed the B/A intensity ratio by normalizing each B-site intensity to the mean of its four nearest A-site neighbors (Fig. 5, D to F). The resulting ratio maps show a broad band of enhanced B/A intensity ratios concentrated at and extending several unit cells along the BTO/LSMO interface (fig. S2). Regions deeper in LSMO display uniform ratios without pronounced anomalies, underscoring the interfacial character of the effect. Spatial overlay with the unit-cell–resolved polarization map shows that these bright B-site pockets colocalize with zones that maintain a downward polarization under both bias polarities, identifying them as candidates for local pinning centers. In contrast to the Pt/BTO interface, where a continuous V_O_-rich layer produces uniform polarization pinning across the entire interface, the pinning at the BTO/LSMO interface is spatially localized and is only observed in the vicinity of La_Mn_ antisite clusters.

To verify the structural origin of the observed HAADF contrast, we performed image simulations by modeling the substitution of Mn at nominal B-sites with La cations (fig. S3). The simulations successfully reproduce the experimental signature: the occupancy of B-site columns by the heavier La (*Z* = 57) instead of Mn (*Z* = 25) markedly increases the B-column intensity. This contrast assignment confirms that the bright B-site contrast originates from La_Mn_ antisites, where the bright B-site pockets reflect the presence of these heavier cation species. While HAADF intensity analysis alone cannot entirely decouple thickness or channeling effects, the assignment of these structural anomalies as La_Mn_ antisites is further corroborated by the localized strain analysis and EELS elemental mapping.

To investigate the structural impact of these defects, we performed strain mapping referenced to a defect-free region of the LSMO electrode. While the in-plane strain component (ε*_xx_*) remains comparatively weak (Fig. 5, G to I), the resulting maps reveal localized tensile strain normal to the interface (ε*_yy_*) concentrated specifically around the La_Mn_ antisite-rich regions (Fig. 5, J to L), consistent with anisotropic strain accommodation under epitaxial constraint. This strain originates from the size and bonding mismatch that occurs when a larger La cation occupies a nominal Mn site, which derives local octahedral distortions and generates defect dipoles (*37*). A flexoelectric contribution may also arise from the strain gradient, further reinforcing the defect-dipole response. These dipoles generate built-in local fields that bias neighboring unit cells, an effect further strengthened when coupled with V_O_s or other charge-compensating defects (*34*, *52*). As a result, the polarization in the BTO unit cells adjacent to the antisite clusters remains oriented downward, forming localized regions that resist reversal even under external fields sufficient to switch the more stoichiometric portions of the film. These results suggest that the La_Mn_ antisites, in conjunction with the observed tensile strain, define a localized pinning landscape at the BTO/LSMO interface.

### Chemical confirmation of antisites and local redox near the interface

**Fig. 6.**
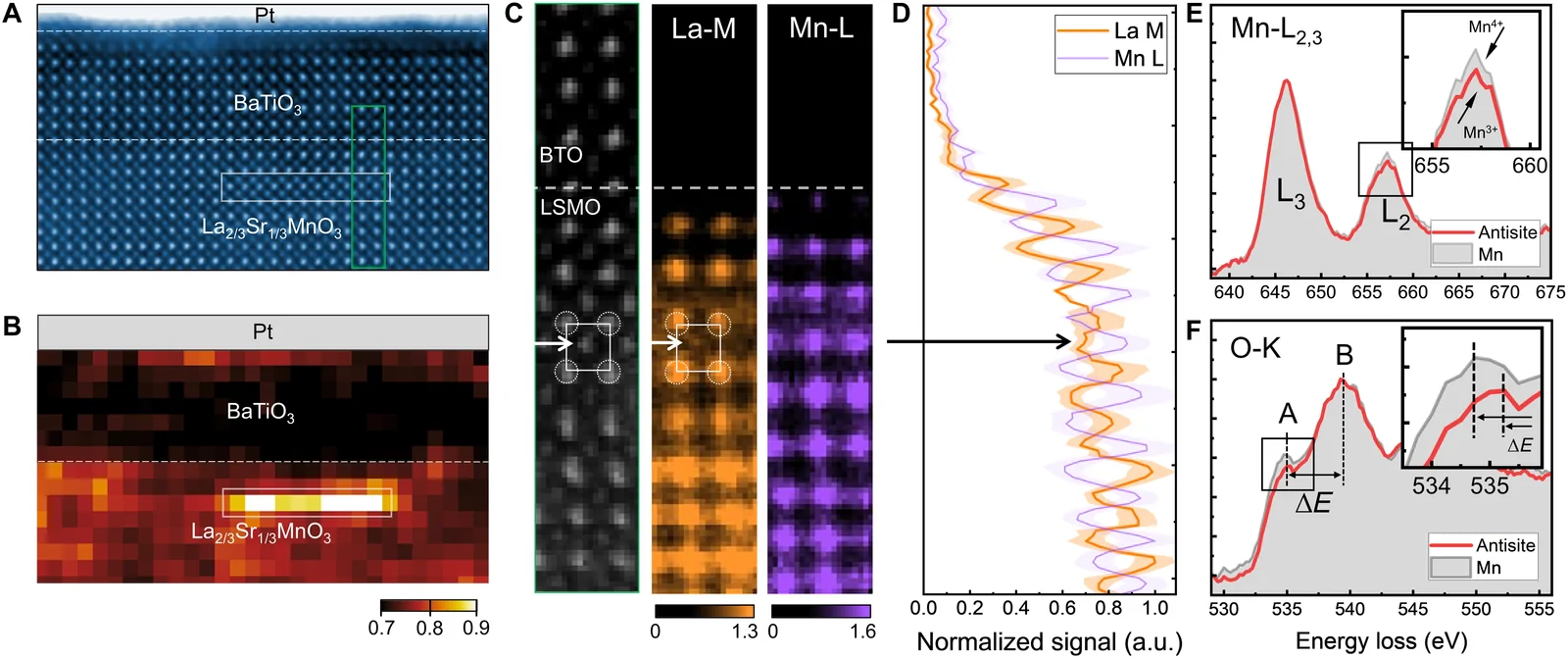
STEM-EELS characterization of La_Mn_ antisites and local redox at the BTO/LSMO interface. (**A**) HAADF-STEM image of the Pt/BTO/LSMO heterostructure. The image is reproduced from Fig. 5B to indicate the region selected for EELS analysis. (**B**) Normalized B/A intensity ratio map derived from (A); the image is reproduced from Fig. 5E. The white rectangles in (A) and (B) mark the antisite-rich region that was prioritized for detailed EELS analysis. (**C**) Atomic-resolution ADF-STEM and EELS elemental maps (La-M_4,5_ and Mn-L_2,3_ edges) acquired from the area indicated by the green box in (A). The white arrow indicates La_Mn_ antisite. (**D**) Site-specific EELS line profiles for La-M and Mn-L signals, extracted across the interface through the antisite shown in (C). The black arrow identifies localized La signal on a nominal B-site column, confirming the cation exchange at the atomic scale. (**E**) Mn-L_2,3_ edge spectra from antisite-rich (red) and stoichiometric (gray) regions. The increased L_3_/L_2_ intensity ratio (inset) signifies a reduced Mn valence state. (**F**) O-K edge spectra; the decreased Δ*E* = B – A near antisites reflects Mn reduction and oxygen loss, consistent with the orbital hybridization shifts at the BTO interface.

To characterize the chemical identity of these pinning sites and the associated electronic environment, we performed site-specific STEM-EELS analysis across the BTO/LSMO interface. We correlated the Z-contrast features with chemical composition using STEM-EELS acquired across BTO and into LSMO, targeting areas with elevated B/A intensity ratios (Fig. 6, A and B). Elemental maps constructed from the La-M_4,5_ and Mn-L_2,3_ edges (Fig. 6, C and D) show that, in regions with bright B-site columns, La signals are strongly enhanced at nominal B-site positions, providing direct evidence for La_Mn_ antisite formation at the LSMO side of the interface (*53*).

To assess accompanying changes in electronic structure, we analyzed fine structures of the Mn-L_2,3_ and O-K edges. In the Mn-L_2,3_ spectra (Fig. 6E), the L_3_/L_2_ intensity ratio increases within the nonstoichiometric zones, consistent with a reduction of Mn valence (enhanced Mn^3+^ fraction) associated with oxygen deficiency (*54*, *55*). The O-K edge fine structure (Fig. 6F) further corroborates this Mn reduction through modified orbital hybridization. The pre-edge feature (peak A; O 2*p*–Mn 3*d* states) exhibits a pronounced energy shift relative to the stable La/Sr-derived peak B (O 2*p*–La 5*d*/Sr 4*d* states), the latter of which serves as an internal energy reference (*56*). The measured decrease in the peak separation (Δ*E* = B – A) near the antisite clusters confirms local oxygen deficiency, where the shift of peak A is associated with modifications in the local electronic and coordination environment under a reduced Mn valence (*57*). These structural and spectroscopic trends point to an interfacial electrochemical redox process, wherein oxygen migration out of the LSMO facilitates both Mn reduction and the energetic stabilization of La_Mn_ antisites. Such chemical reconfiguration, coupled with the localized strain fields, establishes a comprehensive microscopic origin for the inhomogeneous polarization pinning observed at the LSMO interface.

**Fig. 7.**
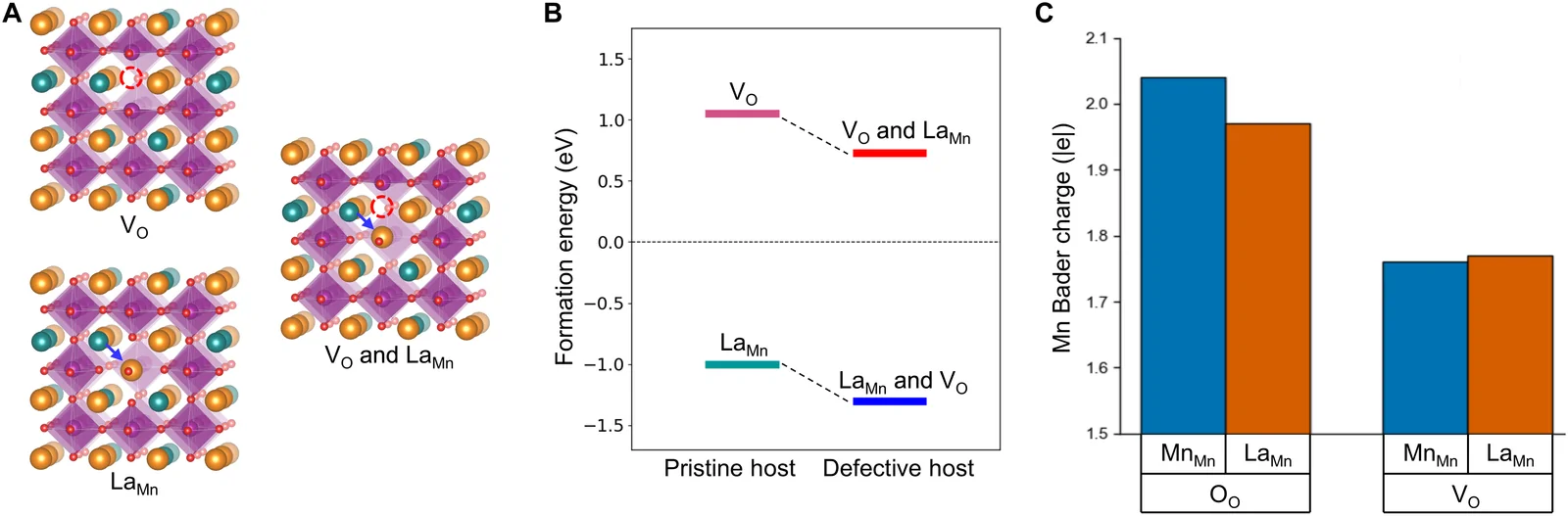
DFT analysis of defect energetics and electronic structure in the LSMO electrode. (**A**) Relaxed atomic models of LSMO supercells with point defects, including an oxygen vacancy (V_O_), a La-on-Mn antisite defect (La_Mn_), and a coexisting V_O_-La_Mn_ complex. (**B**) Calculated formation energies demonstrating the mutual stabilization of defects. The formation energy of a V_O_ is reduced in the presence of a La_Mn_ (top), and conversely, the formation energy of a La_Mn_ is lowered when a V_O_ is present (bottom). (**C**) Mn Bader charge near defect sites calculated for different defect configurations. The presence of V_O_ reduces the Mn charge, whereas the La_Mn_ alone has a negligible effect on the Mn valence. This confirms that the experimentally observed Mn reduction is primarily driven by oxygen deficiency.

To probe the energetic driving forces and electronic consequences of these defects, we performed DFT calculations on the LSMO with these defects (Fig. 7A). Calculations of defect formation energies reveal a strong energetic coupling between La_Mn_ antisite and V_O_s (Fig. 7B). As shown in the formation energy diagram, the energy cost to create a V_O_ is substantially lowered (from ∼1.0 eV to ∼0.75 eV) in a host lattice that already contains a La_Mn_ antisite. Conversely, the presence of a V_O_ makes the formation of a La_Mn_ antisite more favorable, lowering its formation energy (from ∼−1.0 eV to ∼−1.3 eV). This mutual stabilization mechanism provides a clear energetic basis for the costabilization of V_O_s and La_Mn_ antisites observed experimentally.

Furthermore, Bader charge analysis was used to quantify the electronic impact of these V_O_ and La_Mn_ defects. The calculations demonstrate that the presence of a V_O_ is the dominant factor inducing a pronounced reduction in the local Mn valence. As shown in Fig. 7C, the local Mn Bader charge in a structure without vacancies is +2.04 |*e*|. This is only slightly reduced to +1.97 |*e*| when a La_Mn_ antisite is introduced. In contrast, the introduction of a V_O_ causes a remarkable reduction in the Mn charge, dropping it to +1.76 |*e*| (without La_Mn_) and +1.77 |*e*| (with La_Mn_). These results indicate that the La_Mn_ antisite itself is electronically similar to the Mn cation it replaces, whereas the V_O_ is responsible for the large local charge difference. This computational evidence supports the experimental EELS data showing Mn valence reduction in oxygen-deficient interfacial regions.

### Integrated mechanism and implications for FTJ performance

The combined structural and spectroscopic evidence establishes an intrinsically asymmetric interfacial pinning mechanism in the Pt/BTO/LSMO FTJ. At the Pt/BTO interface, a V_O_-rich interfacial layer forms and persists under applied voltage bias, generating a positive space charge that stabilizes a continuous and robust downward polarization across the entire interface and resists reversal under an opposing field. In contrast, at the BTO/LSMO interface, V_O_-assisted La_Mn_ antisites form locally, concentrate tensile out-of-plane strain, and generate internal fields that pin the adjacent BTO polarization downward only in spatially localized regions, while neighboring regions remain comparatively field-responsive. Under upward electric field, interior and LSMO-proximal layers can partially reverse, whereas pinned regions at both interfaces remain downward, forcing the formation of head-to-head domain walls within the ultrathin barrier. These charged walls locally degrade the tunneling barrier, narrowing the effective switching volume and contributing to the nonideal device performance. Such an asymmetric dual-pinning landscape suppresses complete polarization reversal and provides a direct microscopic origin for the limited TER observed in practical FTJ devices.

## DISCUSSION

By combining atomic-resolution imaging, spectroscopy, and in situ polarization mapping, we identify a unified microscopic origin of frustrated polarization switching in Pt/BTO/LSMO FTJ. At the Pt/BTO interface, an oxygen vacancy–rich and chemically reduced layer produces a positive space charge that strongly stabilizes polarization pointing downward. Under an opposing upward electric field, the interior of the ultrathin BTO can switch while the pinned interfacial layers do not, forcing the formation of charged head-to-head domain walls that degrade the effective tunneling barrier and narrow the achievable TER window. While downward-preferred polarization in BaTiO_3_ heterostructures has previously been attributed to V_O_ pinning (*20*–*23*), work-function asymmetry (*24*, *25*), or strain gradients (*27*, *28*), our direct atomic-scale observations confirm this state fundamentally originates from the oxygen-deficient interfacial layer.

Conversely, at the BTO/LSMO interface, V_O_-assisted La_Mn_ antisite clusters generate localized tensile strain field that further pin the adjacent BTO layers. Because their charge is screened by the metallic electrode, the downward direction of this mechanically pinned layer is also influenced by the top-interface electrostatic field. While this local mechanical pinning is consistent with effects from substrate strain (*31*), our results show that the pinning direction is governed by the combined effects of interfacial chemistry, strain, device geometry, and electrostatic boundary conditions (*29*, *30*).

These results suggest concrete routes to recover reversibility and enhance device performance. Reducing oxygen deficiency at both interfaces—through higher oxygen activity during growth, postgrowth reoxidation steps, or insertion of diffusion/chemistry-stabilizing interlayers—should suppress the continuous pinning layer at Pt/BTO and the localized defect/strain pockets at BTO/LSMO. More broadly, controlling coupled anion and cation defect populations provides an actionable framework for engineering oxide interfaces at the unit-cell limit.

## MATERIALS AND METHODS

### Sample growth

Pt, BaTiO_3_ (BTO), and La_2/3_Sr_1/3_MnO_3_ (LSMO) thin films were grown epitaxially on a SrTiO_3_ (STO) (001) substrate (CrysTec GmbH) using pulsed laser deposition with in-situ reflection high-energy electron diffraction (RHEED) monitoring. A 115 nm-thick LSMO layer was grown on the STO substrate. After 8–unit cell thickness of BTO layer grown on the LSMO layer, the sample was annealed at 750°C for 3 hours. After the annealing, piezoelectric force microscopy) (PFM) was performed for poling the BTO layer. The thickness of the BTO and LSMO layers was controlled by monitoring RHEED oscillations. A 20-nm-thick Pt electrode was then deposited on the the BTO layer. Cross-sectional samples for in situ (S)TEM biasing experiments were prepared on a MEMS chip using Ga^+^ ion beam milling at an accelerating voltage from 30 kV down to 5 kV with a dual-beam FIB system (Helios G4, Thermo Fisher Scientific).

### Polarization measurement using STEM-HAADF images

For HAADF-STEM imaging, a detector collection angle in the range of 70 to 200 mrad was used, and the convergence semiangle of the focused probe was 21.5 mrad. Scan distortion was corrected by image correlation with a 90°-rotated image acquired immediately after each scan. The information from the fast-scan direction in each image was used as a reference to correct the distortion along the slow-scan direction, and this process was iteratively repeated until the difference between the two images converged. In the STEM-HAADF images, the positions of individual atomic columns were determined by calculating the center-of-mass of each column intensity through an iterative fitting procedure. The center of each perovskite unit cell was defined using the positions of the A-site cations (Ba), and the displacement of the B-site cation (δ_B_, Ti) was measured relative to this center. Because the contrast of oxygen columns is too weak to be directly resolved in HAADF images, the displacement of the oxygen octahedral center (δ_O_) was estimated from δ_B_ using a proportionality constant κ = −2, as expressed by

$$\delta_{O}={\kappa \delta }_{B}$$ (1)

We assumed κ = −2 based on previous studies on perovskite ferroelectrics, where the displacement of the oxygen octahedral cage center was approximately twice that of the B-site cation (*58*). The adopted Born effective charges account for the anomalously large polarizability of Ti in the BaTiO_3_ lattice and therefore differ substantially from formal ionic charges. The ionic polarization (*P*) of the BaTiO_3_ layer was calculated using

$$P=\frac{1}{V}\sum_{i}\delta_{i}Z_{i}=\frac{1}{V}\left(\delta_{B}Z_{B}+3\delta_{O}Z_{O}\right)=\frac{1}{V}\delta_{B}\left(Z_{B}+3\kappa Z_{O}\right)$$ (2)

where *Z_i_* denotes the Born effective charge of ion *i*. In this study, the following effective charges were used: *Z*_Ba_ = +2.75, *Z*_Ti_ = +7.16, *Z*_O1_ = −2.15, and *Z*_O2_ = −5.71.

### STEM image simulation

STEM image simulations were performed using qSTEM software to evaluate the atomic-column contrast associated with antisite configurations. A model structure of La_2/3_Sr_1/3_MnO_3_ (LSMO) with a thickness of 10-unit cells was constructed using VESTA. To introduce antisite defects, La atoms were systematically substituted at Mn (B-site) positions within the LSMO lattice. The structure was oriented along the [100] zone axis to match the experimental HAADF imaging condition. STEM-HAADF images were simulated using 20 multislices with a sampling size of 0.125 Å/pixel, a probe convergence semiangle of 21.5 mrad, and a detector collection angle of 70 to 200 mrad under an accelerating voltage of 300 kV. Rather than reproducing the exact experimental LSMO thickness, the 10–unit cell model was designed to capture the trend in B-site intensity variation as the number of La-on-Mn substitutions increased. The simulations were therefore intended as a qualitative reference for interpreting the experimentally observed contrast modulation, not as a quantitative reproduction. The resulting HAADF-STEM images were analyzed by measuring the relative intensity of the B-site atomic columns to assess how antisite formation modifies the image contrast.

### EELS data acquisition

Following methods previously reported for EELS acquisition and Ti valence analysis (*18*), aberration-corrected STEM (JEM-ARM300CF, JEOL), equipped with an energy filter (Gatan Quantum ER965), was used to analyze the elemental composition and electronic structure of BTO and LSMO. For elemental mapping, EELS 2D array data from the BTO film and LSMO electrode were recorded over an energy range of 375 to 875 eV, covering the Ba-M_4,5_, Ti-L_2,3_, Mn-L_2,3_, La-M_4,5_, and O-K edges. The energy dispersion and resolution were set to 0.25 eV/pixel and 1.25 eV, respectively. For electronic structure analysis, EELS 2D array data were recorded in the 413 to 612 eV range, focusing on the Ti-L_2,3_, O-K, and Mn-L_2,3_ edges, with an energy dispersion of 0.1 eV/pixel and a resolution of 0.6 eV. For MLLS fitting, reference spectra for Ti^4+^ and Ti^3+^ were obtained from bulk BaTiO_3_ from LaTiO_3_, respectively (*45*). The dwell time for both elemental mapping and electronic structure analysis was set to 0.02 s to minimize specimen drift and prevent electron beam damage. The electron dose rate was maintained at approximately 10^6^ e^−^ nm^−2^ s^−1^, ensuring no substantial damage during data collection.

### DFT calculation

All DFT calculations were performed using the Vienna Ab initio Simulation Package. For the Pt/BaTiO_3_/La_2/3_Sr_1/3_MnO_3_ (Pt/BTO/LSMO) heterostructure, the exchange-correlation energy was treated using the Perdew-Burke-Ernzerhof for solids functional. A plane-wave basis set with a kinetic energy cutoff of 620 eV was used. The model consisted of a slab oriented along the [001] direction, containing a 6–unit cell–thick LSMO BE, a 6–unit cell–thick BTO tunneling barrier, and a TE composed of 12 atomic layers of Pt. To simulate the compressive epitaxial strain from the SrTiO_3_ substrate, the in-plane lattice parameters were constrained to *a* = 3.907 Å. The La/Sr disorder in the electrode was treated using the virtual crystal approximation (VCA). Ferroelectric switching was simulated by displacing the Ti and O sublattices within the BTO layer from their centrosymmetric positions to establish distinct polarization-up (P↑) and polarization-down (P↓) states. The structures were relaxed using a 2 × 2 × 1 Γ-centered k-point mesh until forces fell below 0.05 eV/Å. For the defect analysis in bulk LSMO, separate spin-polarized calculations were performed using a 3 × 3 × 3 supercell (135 atoms). In this model, the La and Sr cations and antisite defects were explicitly distributed within the supercell lattice, rather than using VCA. These calculations used a plane-wave cutoff of 500 eV and sampled the Brillouin zone using a 2 × 2 × 2 Γ-centered k-point grid. The electronic self-consistency loop was converged to 10^−6^ eV.
