# Supplementary material for: Direct visualization of interfacial defect effects on polarization switching in BaTiO3 tunnel junctions
Source: Sci Adv. 2026 Sep 16;12(38):eaeh5401. doi: 10.1126/sciadv.aeh5401 (PMC13580564; doi:10.1126/sciadv.aeh5401)
Supplement: Supplementary file 1 [file sciadv.aeh5401_sm.pdf]

Science Advances
AAAS

Supplementary Materials for

Direct visualization of interfacial defect effects on polarization switching in $BaTiO_3$ tunnel junctions

Jeehun Jeong *et al.*

Corresponding author: Sang Ho Oh, shoh@kentech.ac.kr

Sci. Adv. **12**, eaeh5401 (2026)
DOI: 10.1126/sciadv.aeh5401

This PDF file includes:

Figs. S1 to S3

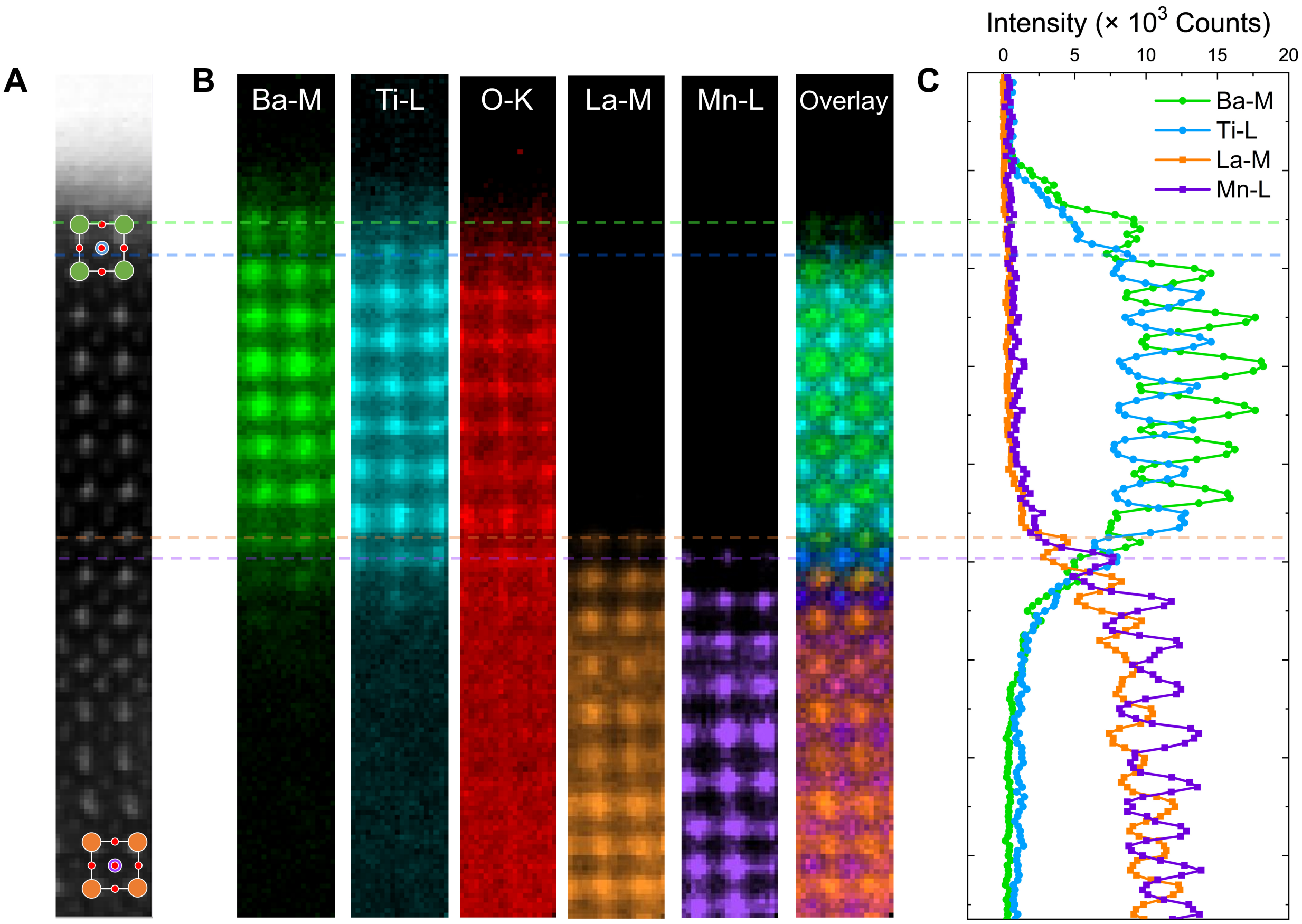

Fig. S1.

Atomic-scale characterization of the two interfaces in the Pt/BTO/LSMO FTJ. (**A**) Virtual ADF image showing the BTO/LSMO interface. (**B**) Corresponding EELS integrated signal maps for Ba-M, Ti-L, O-K, La-M, Mn-L edges, together with cation-overlaid map. (**C**) Line profiles of the cation signals extracted from (**B**), highlighting the elemental variations across the interface. The dotted lines indicate the column terminations at the two interfaces.

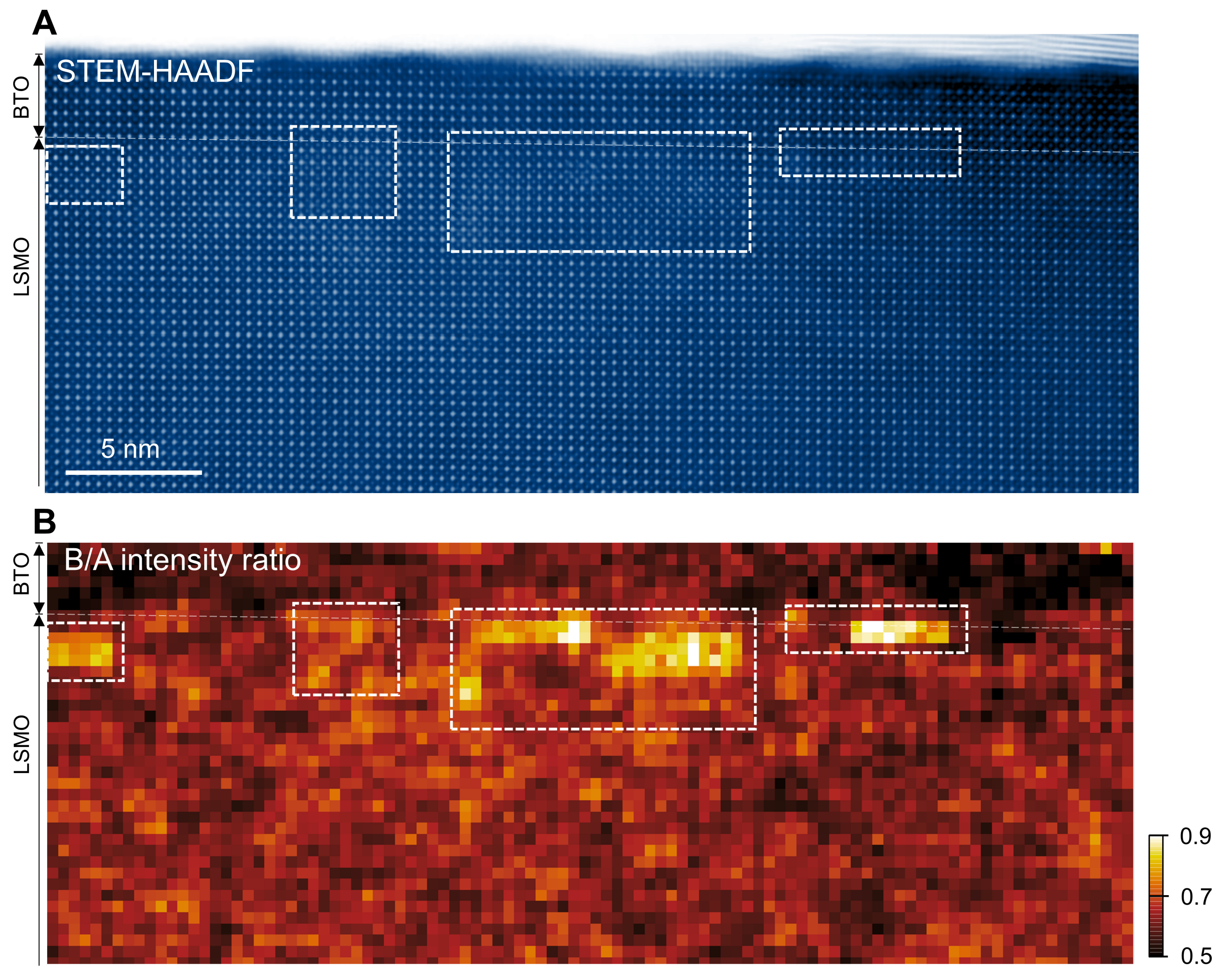

Fig. S2.

Atomic-resolution STEM-HAADF image and corresponding B-site/A-site normalized intensity map of the Pt/BTO/LSMO FTJ. (**A**) STEM-HAADF image resolving the BTO/LSMO interface. (**B**) B-site column intensity normalized to the adjacent A-site columns, highlighting relative cation-site contrast variations. The normalized map shows a pronounced increase in the B-site column intensity near the BTO/LSMO interface, as marked by the white dotted rectangle, consistent with an enhanced presence of A-site cations (La) substituting onto B-site positions (Mn), indicative of antisite defect formation in this interfacial region.

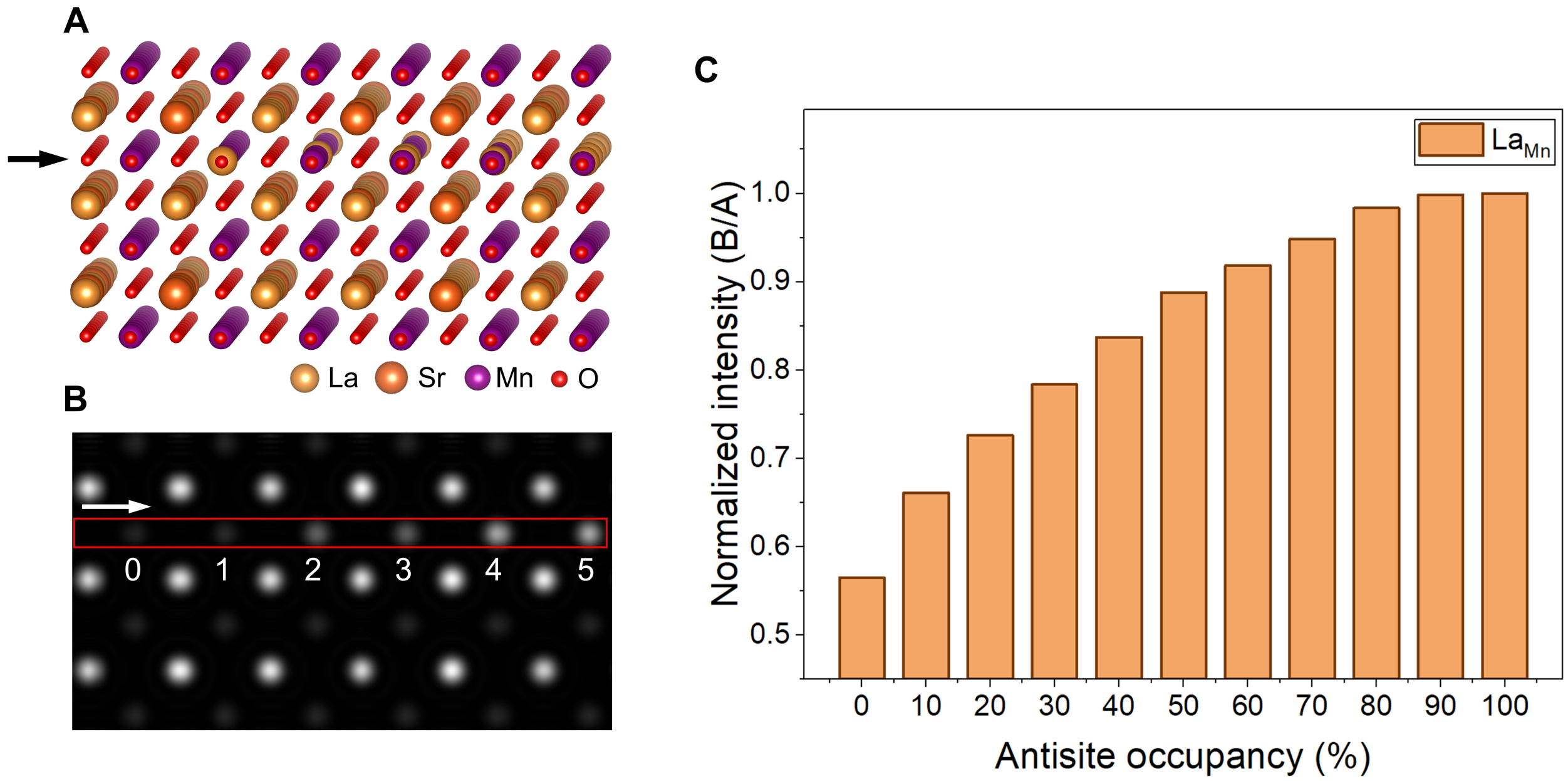

Fig. S3.

STEM-HAADF intensity variation associated with La_{Mn} antisite defects. (**A**) Atomic structure of LSMO, where the row containing La_{Mn} antisite defects is indicated by black arrows. (**B**) Simulated STEM-HAADF images constructed from the structural models in (**A**), with the number of La atoms substituting Mn sites increasing from 0 to 5 from left to right. (**C**) B-site column intensity normalized to the adjacent A-site columns, plotted as a function of the number of La atoms exchanged onto Mn sites (0–10). The simulations show a monotonic increase in the normalized B-site intensity with increasing La-on-Mn substitution, suggesting that La incorporation at Mn sites enhances the HAADF contrast and that such intensity variations can serve as an indirect indicator of antisite defects.
